# The association between constipation and anxiety: a cross-sectional study and Mendelian randomization analysis

**DOI:** 10.3389/fpsyt.2025.1543692

**Published:** 2025-03-31

**Authors:** Yingxuan Huang, Yubin Wang, Boming Xu, Yilin Zeng, Peizhong Chen, Yisen Huang, Xiaoqiang Liu

**Affiliations:** Department of Gastroenterology, First Hospital of Quanzhou Affiliated to Fujian Medical University, Quanzhou, Fujian, China

**Keywords:** constipation, anxiety, American adults, cross-sectional study, NHANES, Mendelian randomization analysis

## Abstract

**Objective:**

The relationship between constipation and anxiety remains underexplored. This study investigates the association between constipation and anxiety in a representative sample of adults in the United States.

**Methods:**

A cross-sectional analysis was conducted using data from the National Health and Nutrition Examination Survey (NHANES) from 2007 to 2010, including 9,126 adults aged ≥20 years. Constipation and anxiety were assessed using standardized survey instruments. Multivariable logistic regression models were employed to calculate adjusted odds ratios (ORs), and subgroup and sensitivity analyses were performed to validate the findings. Additionally, Mendelian randomization (MR) was employed to assess the potential causal relationship between constipation and anxiety using genetic data from large GWAS datasets.

**Results:**

Of the 9,126 participants, 324 reported constipation (prevalence: 3.6%), and 2,424 reported anxiety (prevalence: 26.6%). Anxiety prevalence was significantly higher in individuals with constipation compared to those without (41.4% vs. 26.0%; *P* < 0.001). After adjusting for demographic, socioeconomic, lifestyle, and comorbid factors, constipation remained independently associated with anxiety (adjusted OR: 1.33, 95% CI: 1.02–1.73; *P* = 0.038). Subgroup analyses revealed no significant interactions. Sensitivity analyses, including multiple imputations, weighted analysis, and propensity score matching, corroborated the robustness of the results. MR analysis, however, revealed no significant causal association between constipation and anxiety.

**Conclusion:**

This study identifies a significant association between constipation and anxiety in a large, nationally representative cohort. While the association remains robust after adjusting for various factors, MR did not provide evidence for a causal relationship. Clinicians should consider evaluating and addressing anxiety symptoms as part of a comprehensive management strategy for patients presenting with constipation.

## 1 Introduction

Anxiety is a common mental health disorder characterized by persistent worry, tension, and various physical symptoms such as palpitations, sweating, and shortness of breath (1). The prevalence of anxiety disorders is rising globally, significantly impacting individuals’ quality of life and social functioning. A prospective cohort study based on Danish national registry data found that anxiety patients had a 39% increased risk of natural mortality and a 146% increased risk of unnatural mortality (2). Thus, screening and early intervention for anxiety disorders are crucial, with identifying risk factors and high-risk populations being key components.

Constipation is another prevalent health issue, often presenting as reduced bowel movement frequency, difficulty in defecation, and incomplete evacuation (3). The prevalence of constipation varies across populations but is generally common among adults. Constipation not only affects physical health but also significantly impacts patients’ quality of life, causing discomfort and psychological distress (4).

Existing research has increasingly revealed the close link between constipation and anxiety. For instance, a study evaluating social anxiety symptoms in children with chronic functional constipation found a prevalence of social anxiety of 67.5%, with a significantly higher rate and severity among girls (5). Additionally, a study in a Chinese population aged 60 and above identified anxiety as a risk factor for constipation. However, most of these studies focus on adolescents or the elderly, with relatively few addressing adults. Moreover, most existing studies are based on specific ethnic or regional populations, leaving it unclear whether these findings apply to other races or broader populations (6).

This study seeks to explore the relationship between constipation and anxiety, focusing on the adult population in the United States. Given the uncertain nature of this association, we aim to assess its causal link using Mendelian randomization (MR). By analyzing existing datasets, we aim to clarify the association between constipation and anxiety and provide a foundation for developing targeted psychological interventions for individuals affected by constipation.

## 2 Methods

### 2.1 Data source

**Figure 1:**
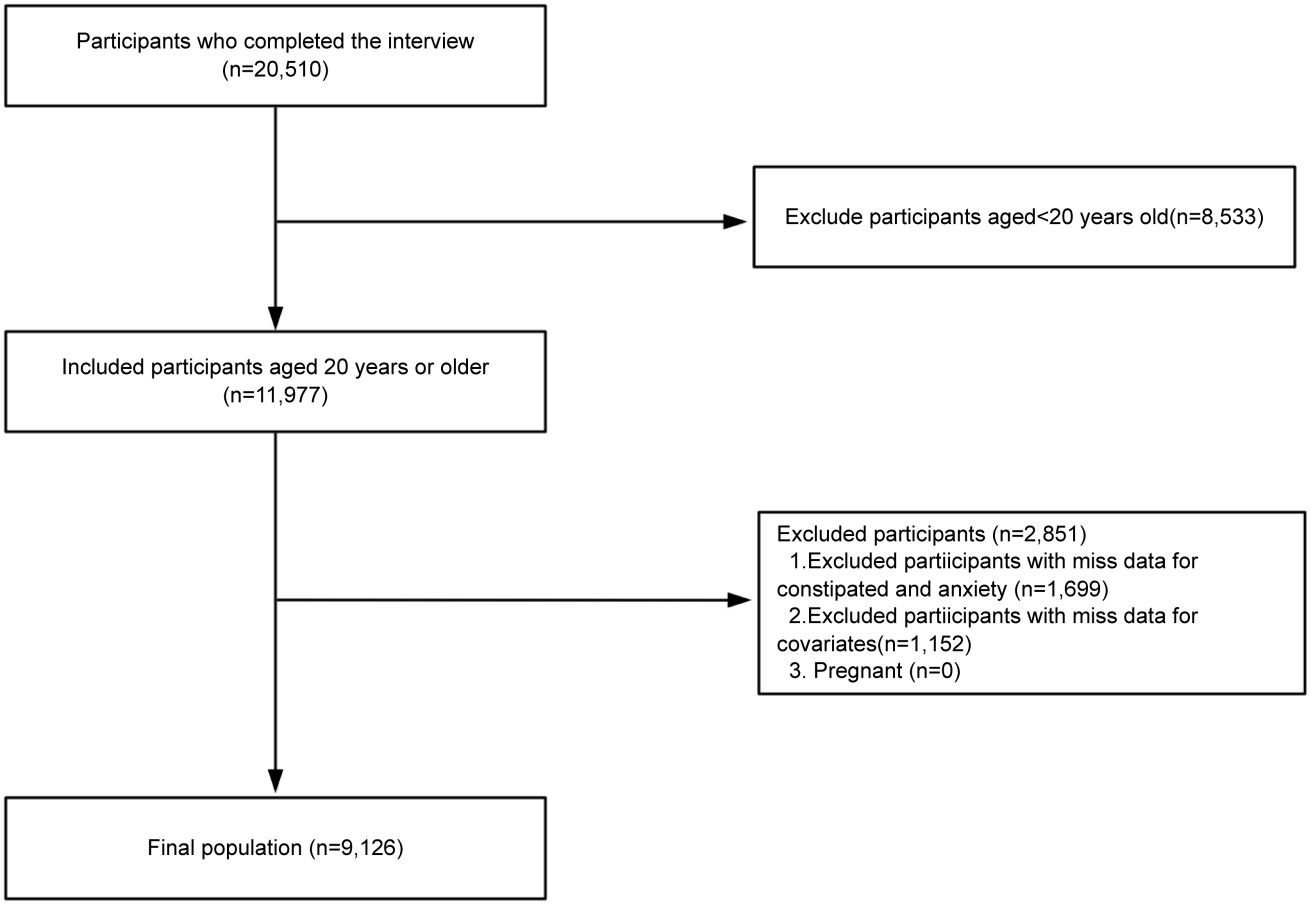
Study flow chart.

Data for this study were sourced from the National Health and Nutrition Examination Survey (NHANES) conducted between 2007 and 2010. The inclusion of data from only these cycles is since the specific questionnaire on constipation was only available in the NHANES surveys during these two periods, which provided the relevant data for this analysis. NHANES, conducted by the National Center for Health Statistics (NCHS) of the Centers for Disease Control and Prevention (CDC), aims to assess the health and nutritional status of the non-institutionalized civilian population of the United States. Data collection includes demographic, socioeconomic, and health-related interviews during home visits, along with physical examinations and laboratory assessments conducted in mobile examination centers (MECs). From the 2007-2010 NHANES cycles, we selected 11,977 adults aged 20 years and above. Individuals lacking data on anxiety, constipation, or covariates were excluded. Ultimately, 9,126 participants were included in the analysis. (**Figure 1**) Approval for the NHANES study protocol was granted by the NCHS Research Ethics Review Board, with participants providing written informed consent. The use of publicly available deidentified data eliminated the need for additional consent. This study complied with the STROBE reporting guidelines.

### 2.2 Definition of constipation

Constipation was assessed using the bowel health questionnaire in NHANES, defined as having fewer than three bowel movements per week. Participants were asked to estimate their weekly bowel movement frequency, with fewer than three times per week classified as constipation, three to 21 times as normal, and more than 21 times as diarrhea (7).

### 2.3 Assessment of anxiety

Anxiety was evaluated during personal interviews using the question: “How many days during the past 30 days did you feel worried, tense, or anxious?” This assessment is based on the CDC’s 14-item health days measure incorporated into health-related quality of life (HRQoL) evaluations. Anxiety status was categorized as “No” (0 to 6 days per month) and “Yes” (7 to 30 days per month) (8).

### 2.4 Covariates

Based on previous studies (7, 9), this study assessed various potential covariates related to constipation and anxiety, including age, gender, race, marital status, body mass index (BMI), poverty income ratio (PIR), educational level, smoking status, alcohol intake, total physical activity time, cardiovascular disease (CVD), hypertension, diabetes, pulmonary disease, arthritis, liver disease, cancer, and depression. Definitions are as follows: age was a continuous variable; gender was recorded; race was categorized into non-Hispanic White, non-Hispanic Black, Mexican American, other Hispanic, and other races; marital status was divided into married, never married, cohabiting, and other (including widowed, divorced, or separated); BMI was calculated based on height and weight; PIR was categorized into 1-1.3, 1.31-3.50, and >3.50; educational level was divided into less than high school, high school or equivalent, and above high school; smoking status was categorized as never (smoked less than 100 cigarettes in a lifetime), former (smoked over 100 cigarettes but not currently smoking), and current (smoked over 100 cigarettes and currently smoking) (10); alcohol intake was categorized into never (less than 12 drinks in a lifetime), former (at least 12 drinks in a year but not in the last year), and current, with further subcategories for heavy, moderate, and light use (11); total physical activity time was continuous (12); CVD included angina, chronic heart failure, coronary artery disease, or myocardial infarction; hypertension was determined by average systolic BP ≥ 140 mmHg and/or diastolic BP ≥ 90 mmHg, self-reported diagnosis, or use of antihypertensive medication; diabetes was determined by diagnosis, HbA1c levels ≥ 6.5%, fasting glucose levels ≥ 7.0 mmol/L, random/2-hour Oral glucose tolerance test (OGTT) glucose levels ≥ 11.1 mmol/L, or use of diabetes medication/insulin; pulmonary disease included emphysema, asthma, or chronic bronchitis; arthritis was self-reported; liver disease was self-reported; cancer was self-reported; depression was assessed using the PHQ-9 scale, with scores ≥ 10 indicating depression (13).

### 2.5 Mendelian randomization study design

We conducted a two-sample MR analysis to assess the causal relationship between constipation and anxiety. Summary data for constipation GWAS were obtained from FinnGen (https://storage.googleapis.com/finngen-public-data-r11/summary_stats/finngen_R11_K11_CONSTIPATION.gz), including 44,590 cases and 409,143 controls. Summary data for anxiety GWAS were obtained from the UK Biobank (https://pheweb.org/UKB-SAIGE/download/300), including 6,939 cases and 328,930 controls. The FinnGen study was proved by the Coordinating Ethics Committee of the Helsinki and Uusimaa Hospital District (Nr HUS/990/2017). All participants provided written informed consent. UK Biobank received ethical approval from the North West Multi-Centre Research Ethics Committee (REC reference: 16/NW/0274) and was conducted in accordance with the principles of the Declaration of Helsinki.

### 2.6 Selection of genetic instrumental variables

The selected genetic instrumental variables need to satisfy the following three assumptions: (a) the instruments are significantly associated with the exposure (constipation); (b) the instruments influence the outcome (anxiety) only through the exposure; (c) the instruments are independent of any confounders of the exposure and outcome (14).

To determine the instrumental variants (IVs) for constipation, we selected single nucleotide polymorphisms (SNPs) that were significantly associated with constipation in large GWAS studies (P < 5 × 10^−8^, linkage disequilibrium coefficient [LD] r² < 0.001). For each SNP, we calculated R² to assess the proportion of variance in the exposure explained and calculated F-statistics to evaluate the strength of the association between the SNP and the exposure (15). SNPs with F-statistics <10 were excluded to avoid weak instrument bias.

### 2.7 Statistical analysis

Descriptive analyses were conducted for all participants. Continuous variables were presented as means and standard deviations (SD) or medians and interquartile ranges (IQR) for non-normally distributed data, while categorical variables were expressed as percentages. For continuous variables, comparisons between groups were performed using the independent samples t-test, provided the data followed a normal distribution. If the variables were not normally distributed, the Mann-Whitney U test was employed. Chi-square tests were used for categorical variables. Multivariable logistic regression models analyzed the relationship between constipation and anxiety. Unadjusted and various adjusted models were used: Model 1: Adjusted for age and gender. Model 2: Additionally adjusted for race, marital status, BMI, PIR, educational level, smoking status, alcohol intake, and total physical activity time. Model 3: Further adjusted for CVD, hypertension, diabetes, pulmonary disease, arthritis, liver disease, cancer, and depression.

Subgroup and interaction analyses tested the stability of the constipation-anxiety association. Statistical significance was determined by comparing adjusted ORs with 1.0 and describing 95% confidence intervals (CIs). Additionally, several sensitivity analyses assessed the robustness of the results. First, multiple imputations based on five replicates addressed missing covariate data. Second, NHANES guidelines recommend using sampling weights and design variables to avoid biased estimates and exaggerated significance levels, so complex sampling designs and weights were used in analyses. Third, propensity score matching (PSM) employed a 1:1 nearest neighbor matching algorithm with a caliper width of 0.2. Variables used to generate propensity scores were identical to Model 3. The extent of PSM was checked using standardized mean differences, with <0.1 considered an acceptable threshold.

In the MR analysis, the inverse variance weighted (IVW) method was used as the primary method to explore the relationship between constipation and anxiety. Various methods such as MR-Egger, weighted median, simple mode, and weighted mode were applied to test the reliability and stability of the results. All statistical analyses were conducted using R statistical software (version 4.2.1; http://www.R-project.org) and Free Statistics software (version 1.7; Beijing, China, http://www.clinicalscientists.cn/freestatistics), with the “TwoSampleMR” (version 0.5.6) and “MendelianRandomization” (version 0.5.1) packages used in the R environment.

## 3 Results

### 3.1 Baseline characteristics

In **Table 1**, Among the 9,126 participants, 324 reported constipation (3.6% prevalence), and 2,424 reported anxiety (26.6% prevalence). The average age in the anxiety group was 47.3 years (SD 16.1), significantly lower than the non-anxiety group’s 50.9 years (SD 18.1) (*P* < 0.001). The proportion of females was significantly higher in the anxiety group (59.4%) compared to the non-anxiety group (46.6%) (*P* < 0.001). Other baseline characteristics, including race, marital status, BMI, PIR, educational level, smoking status, and alcohol intake, also differed significantly between the anxiety and non-anxiety groups.

**Table 1:** Baseline characteristics of participants in the NHANES 2007–2010 cycle.

Variables	Total (n = 9126)	No Anxiety (n = 6702)	Anxiety (n = 2424)	Test of significance
Age, Mean ± SD	50.0 ± 17.7	50.9 ± 18.1	47.3 ± 16.1	T, *P*< 0.001
Gender, n (%)				χ ^2^, *P*< 0.001
Male	4561 (50.0)	3576 (53.4)	985 (40.6)	
Female	4565 (50.0)	3126 (46.6)	1439 (59.4)	
Race, n (%)				χ ^2^, *P* =0.011
Non-Hispanic White	4580 (50.2)	3332 (49.7)	1248 (51.5)	
Non-Hispanic Black	1732 (19.0)	1317 (19.7)	415 (17.1)	
Mexican American	1536 (16.8)	1132 (16.9)	404 (16.7)	
Other Hispanic	904 ( 9.9)	636 (9.5)	268 (11.1)	
Other Race	374 ( 4.1)	285 (4.3)	89 (3.7)	
Marital status, n (%)				χ ^2^, *P* < 0.001
Married	4807 (52.7)	3680 (54.9)	1127 (46.5)	
Never married	1508 (16.5)	1086 (16.2)	422 (17.4)	
Living with partner	697 ( 7.6)	470 (7.0)	227 (9.4)	
Other	2114 (23.2)	1466 (21.9)	648 (26.7)	
BMI (kg/m^2^), Mean ± SD	29.1 ± 6.8	29.0 ± 6.6	29.5 ± 7.2	T, *P*=0.001
PIR group1, n (%)				χ ^2^, *P* < 0.001
1-1.3	2839 (31.1)	1914 (28.6)	925 (38.2)	
1.31-3.50	3495 (38.3)	2599 (38.8)	896 (37.0)	
>3.50	2792 (30.6)	2189 (32.7)	603 (24.9)	
Education level, n (%)				χ ^2^, *P* < 0.001
Less than high school	2530 (27.7)	1777 (26.5)	753 (31.1)	
High school or equivalent	2183 (23.9)	1655 (24.7)	528 (21.8)	
Above high school	4413 (48.4)	3270 (48.8)	1143 (47.2)	
Smoking status, n (%)				χ ^2^, *P* < 0.001
Never	4760 (52.2)	3617 (54.0)	1143 (47.2)	
Former	2313 (25.3)	1781 (26.6)	532 (21.9)	
Current	2053 (22.5)	1304 (19.5)	749 (30.9)	
Alcohol intake, n (%)				χ ^2^, *P* < 0.001
Never	1190 (13.0)	926 (13.8)	264 (10.9)	
Former	1774 (19.4)	1278 (19.1)	496 (20.5)	
Current	6162 (67.5)	4498 (67.1)	1664 (68.6)	
PA total time, Median (IQR)	260.0 (0.0, 1020.0)	287.5 (0.0, 1050.0)	210.0 (0.0, 960.0)	U, *P*< 0.001
CVD, n (%)				χ ^2^, *P* =0.007
No	8098 (88.7)	5983 (89.3)	2115 (87.3)	
Yes	1028 (11.3)	719 (10.7)	309 (12.7)	
Hypertension, n (%)				χ ^2^, *P* =0.579
No	5246 (57.5)	3841 (57.3)	1405 (58)	
Yes	3880 (42.5)	2861 (42.7)	1019 (42.0)	
Diabetes, n (%)				χ ^2^, *P* =0.397
No	7450 (81.6)	5485 (81.8)	1965 (81.1)	
Yes	1676 (18.4)	1217 (18.2)	459 (18.9)	
Pulmonary disease, n (%)				χ ^2^, *P* < 0.001
No	7505 (82.2)	5687 (84.9)	1818 (75.0)	
Yes	1621 (17.8)	1015 (15.1)	606 (25.0)	
Arthritis, n (%)				χ ^2^, *P* < 0.001
No	6523 (71.5)	4914 (73.3)	1609 (66.4)	
Yes	2603 (28.5)	1788 (26.7)	815 (33.6)	
Liver disease, n (%)				χ ^2^, *P* < 0.001
No	8817 (96.6)	6528 (97.4)	2289 (94.4)	
Yes	309 ( 3.4)	174 (2.6)	135 (5.6)	
Cancer, n (%)				χ ^2^, *P* 0.034
No	8192 (89.8)	5989 (89.4)	2203 (90.9)	
Yes	934 (10.2)	713 (10.6)	221 (9.1)	
Depression, n (%)				χ ^2^, *P* < 0.001
No	8245 (90.3)	6538 (97.6)	1707 (70.4)	
Yes	881 ( 9.7)	164 (2.4)	717 (29.6)	
Constipation, n (%)				χ ^2^, *P* < 0.001
No	8802 (96.4)	6512 (97.2)	2290 (94.5)	
Yes	324 ( 3.6)	190 (2.8)	134 (5.5)	

BMI, Body Mass Index; PIR, poverty income ratio; PA, physical activity; CVD, cardiovascular disease; χ ^2^, Chi-square test; T, T-test; U, Mann-Whitney U test.

Data are expressed as mean ± standard deviation (SD) for normally distributed continuous variables, and median (interquartile range, IQR) for non-normally distributed continuous variables. Categorical variables are expressed as percentages.

### 3.2 Association between constipation and anxiety

In **Table 2**, Multivariable logistic regression models indicated that the unadjusted model showed a significantly increased risk of anxiety among constipation patients (OR: 2.01, 95% CI: 1.6-2.51, *P* < 0.001). After adjusting for gender and age (Model 1), the association remained significant (OR: 1.72, 95% CI: 1.36-2.16, *P* < 0.001). Further adjustment for race, marital status, BMI, PIR, educational level, smoking status, alcohol intake, and total physical activity time (Model 2) showed the association remained significant (OR: 1.63, 95% CI: 1.29-2.06, *P* < 0.001). In the fully adjusted model (Model 3), including CVD, hypertension, diabetes, lung disease, arthritis, liver disease, cancer, and depression, the association remained significant (OR: 1.33, 95% CI: 1.02-1.73, *P* = 0.038).

**Table 2:** Association between constipation and anxiety.

Variable	Non-adjusted		Model 1		Model 2		Model 3	
	OR (95%CI)	*P*-value	OR (95%CI)	*P*-value	OR (95%CI)	*P*-value	OR (95%CI)	*P*-value
Constipated	2.01 (1.6~2.51)	<0.001	1.72 (1.36~2.16)	<0.001	1.63 (1.29~2.06)	<0.001	1.33 (1.02~1.73)	0.038

BMI, Body Mass Index; PIR, poverty income ratio; PA, physical activity; CVD, cardiovascular disease.

Model 1: Adjusted for age and gender.

Model 2: Additionally adjusted for race, marital status, BMI, PIR, educational level, smoking status, alcohol intake, and total physical activity time.

Model 3: Further adjusted for CVD, hypertension, diabetes, pulmonary disease, arthritis, liver disease, cancer, and depression.

### 3.3 Subgroup analysis

**Figure 2:**
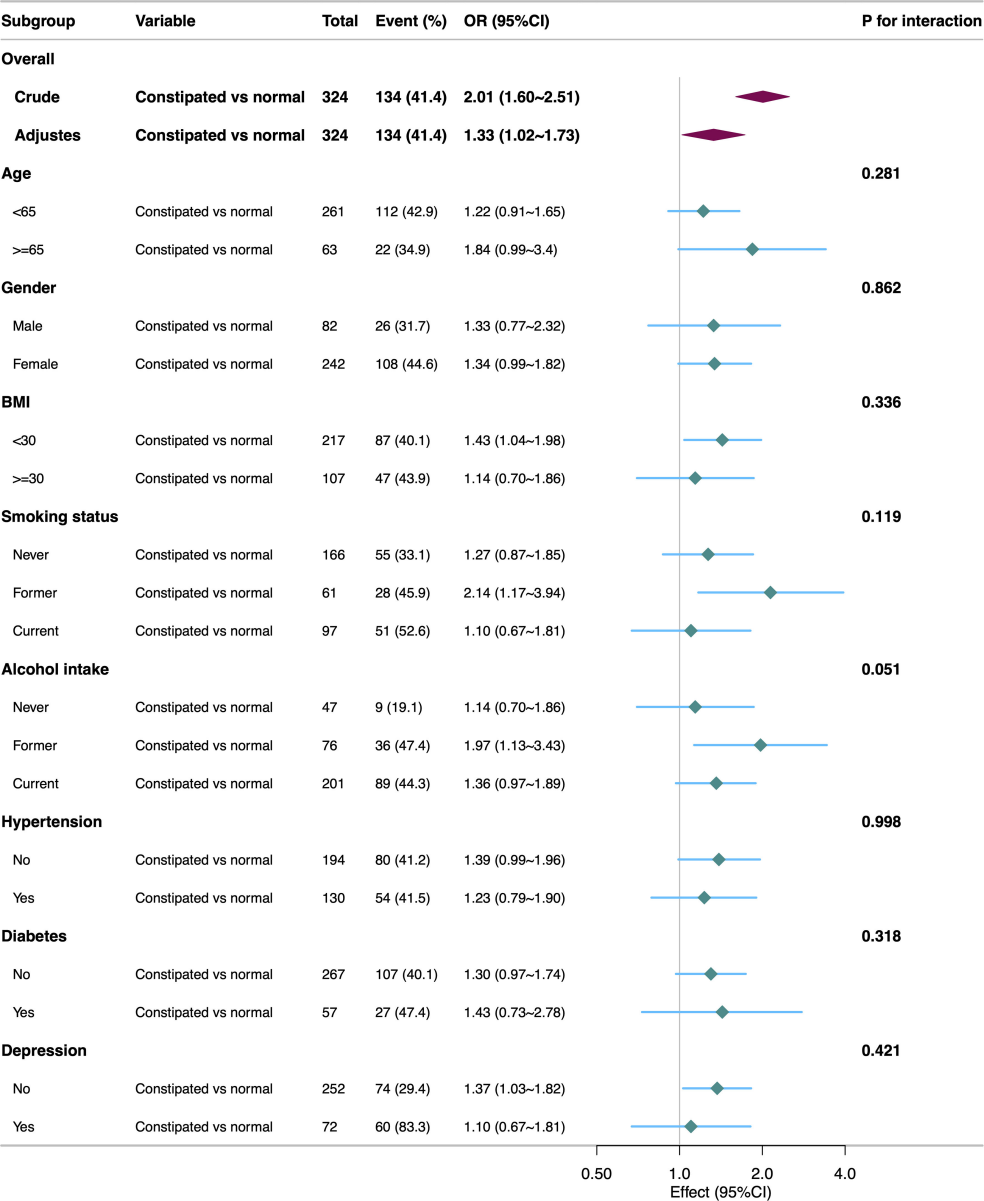
Subgroup analyses for the association of constipation and anxiety.

Subgroup analysis results, displayed in **Figure 2**, showed no statistically significant interactions between constipation and anxiety across age, gender, BMI, smoking status, alcohol intake, hypertension, and diabetes, except (all *P* > 0.05). The association between constipation and anxiety remained relatively stable across all subgroups.

### 3.4 Sensitivity analysis

In **Table 3**, Sensitivity analyses using multiple imputations and weighted analysis indicated that the association between constipation and anxiety remained significant in all models. Multiple imputations showed a significantly increased risk of anxiety among constipation patients (OR: 2.03, 95% CI: 1.64-2.51, *P* < 0.001), remaining significant in the fully adjusted model (OR: 1.34, 95% CI: 1.04-1.73, *P* = 0.023). Weighted analysis showed the association remained significant in all models (OR: 2.01, 95% CI: 1.49-2.73, *P* < 0.001), remaining significant in the fully adjusted model (OR: 1.57, 95% CI: 1.05-2.35, *P* = 0.036). Post-PSM, 324 pairs were well-matched with no significant differences between groups. Post-PSM, the risk ratio for anxiety was 1.47 (95% CI: 1.07-2.03, *P* = 0.018).

**Table 3:** Sensitivity analyses.

Variable	Non-adjusted		Model 1		Model 2		Model 3	
	OR (95%CI)	*P*-value	OR (95%CI)	*P*-value	OR (95%CI)	*P*-value	OR (95%CI)	*P*-value
Multiple imputation	2.03 (1.64~2.51)	<0.001	1.74 (1.4~2.16)	<0.001	1.65 (1.32~2.06)	<0.001	1.34 (1.04~1.73)	0.023
weighted analysis	2.01 (1.49~2.73)	<0.001	1.74 (1.28~2.35)	<0.001	1.6 (1.15~2.22)	0.009	1.57 (1.05~2.35)	0.036
propensity score matching	–	–	–	–	–	–	1.47 (1.07~2.03)	0.018

BMI, Body Mass Index; PIR, poverty income ratio; PA, physical activity; CVD, cardiovascular disease.

Model 1: Adjusted for age and gender.

Model 2: Additionally adjusted for race, marital status, BMI, PIR, educational level, smoking status, alcohol intake, and total physical activity time.

Model 3: Further adjusted for CVD, hypertension, diabetes, pulmonary disease, arthritis, liver disease, cancer, and depression.

### 3.5 Mendelian randomization analysis

**Figure 3:**
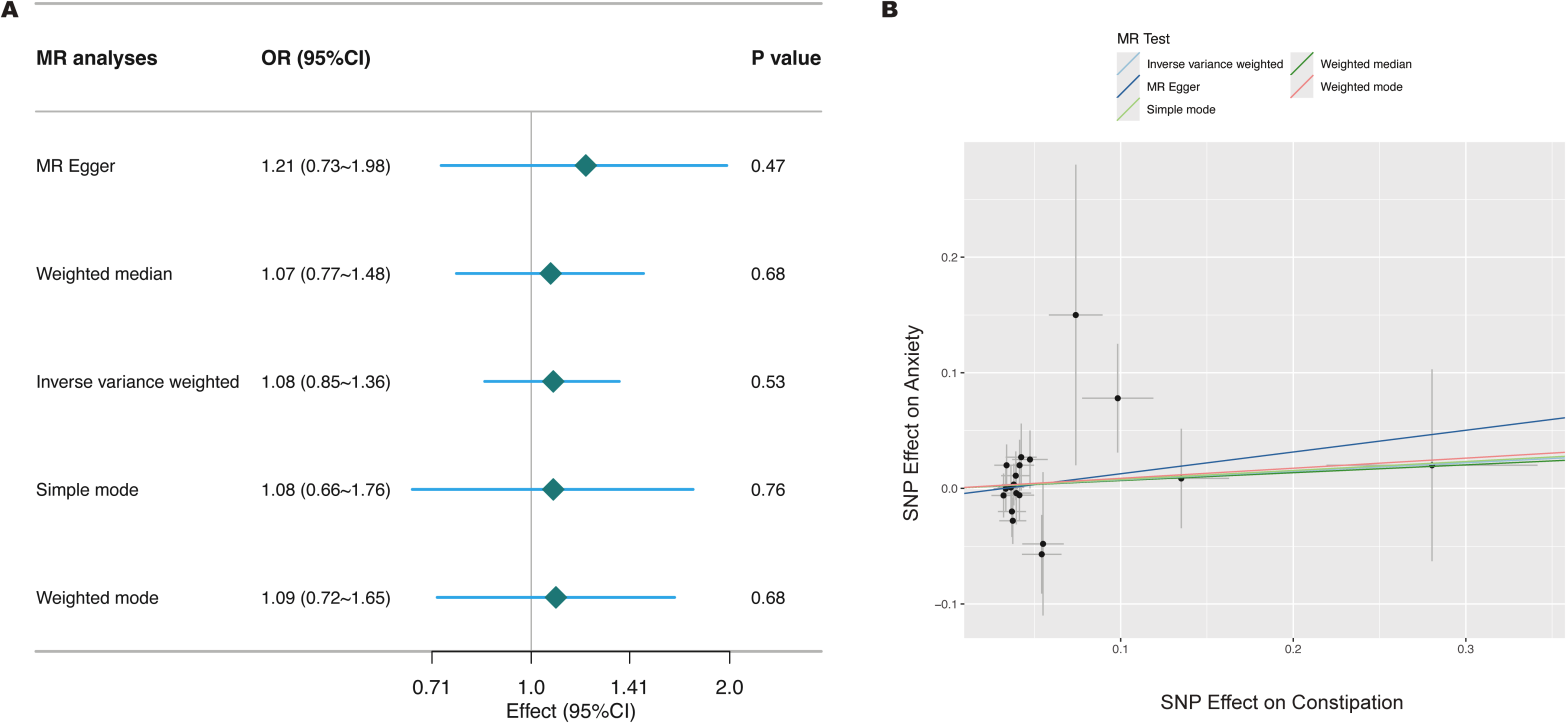
Mendelian Randomization (MR) Plots for relationship of constipation with anxiety **(A)**, Forest plot of five Mendelian Randomization estimators of the effect of constipation on anxiety; **(B)**, Scatter plot of SNPs associated with constipation and the risk of anxiety.

From the GWAS data, 19 independent SNPs significantly associated with constipation were selected as genetic instrumental variables for constipation. All instrumental variables had F-statistics greater than 10, indicating no weak instrument bias. In **Figure 3**, the results from the IVW method showed no significant causal association between constipation and anxiety (OR = 1.08, 95% CI: 0.85–1.36, *P* = 0.53). The weighted median method also yielded a non-significant result (OR = 1.07, 95% CI: 0.77–1.48, *P* = 0.68), and similarly, the weighted mode method showed no significant effect (OR = 1.09, 95% CI: 0.72–1.65, *P* = 0.68). Funnel plot analysis did not reveal significant bias (**Supplementary Figure 1**). Leave-one-out sensitivity analysis showed that the results did not change significantly after removing any SNP, further supporting the robustness of the results (**Supplementary Figure 2**).

## 4 Discussion

This study found a significant positive association between constipation and anxiety. In the unadjusted model, constipation patients had a significantly increased risk of anxiety (OR: 2.01), and this association remained significant after fully adjusting for various covariates (OR: 1.33). Subgroup analysis indicated that the association between constipation and anxiety remained stable across all subgroups. Sensitivity analyses using multiple imputations and weighted analysis confirmed the robustness of the findings. However, the MR analysis showed no evidence of a causal relationship between constipation and anxiety.

Current research on the relationship between constipation and anxiety presents inconsistent findings. Some studies have found associations between constipation and mental health issues like anxiety and depression, while others have not confirmed these associations. For instance, a study in the general Asian population found that constipation related to anxiety and depression was common, with regression analysis indicating that females and high anxiety levels were independent predictors of perceived constipation (16). Another multicenter cross-sectional study found that the prevalence of functional constipation among medical students was 26.3%, with female students, those with severe anxiety, and irregular breakfast eaters more likely to experience constipation (17). These findings align with our study, showing lower mean age and higher female proportion in the anxiety group. These results suggest that younger individuals and females might be more susceptible to anxiety. Clinical evaluation and treatment of constipation patients should focus on these high-risk groups for early identification and management of anxiety symptoms. Our study also found higher proportions of participants with CVD, lung disease, arthritis, and liver disease in the anxiety group. These chronic diseases are significantly associated with anxiety. Chronic diseases might affect patients’ mental health through various mechanisms, exacerbating anxiety and constipation symptoms. This suggests the necessity of considering overall health status and psychological condition when managing constipation patients to achieve better treatment outcomes. Additionally, constipation patients often exhibit fecal urgency. Research indicates that the presence of anxiety is an independent predictor of moderate to severe fecal urgency (18).

In a study of Korean high school students, constipation was not significantly associated with anxiety but was significantly associated with depression (19). However, constipation in this study was defined solely based on stool consistency in the Bristol Stool Form Scale, excluding bowel movement frequency. Bowel movement frequency is a crucial feature of constipation (20), used as a valid method in NHANES datasets (21). Research indicates a low correlation between stool consistency and bowel movement frequency (22). Therefore, incorporating bowel movement frequency in the definition of constipation is necessary for a comprehensive study of the relationship. Another study found significant associations between constipation and depression in Parkinson’s disease patients, but not with anxiety after adjusting for other variables (23). These inconsistent findings might result from different definitions of constipation or anxiety, small sample sizes, short follow-up periods, and insufficient consideration of confounding factors. Our study overcame these limitations by using a large NHANES sample, comprehensive covariate adjustments, and various sensitivity analyses, providing more robust evidence supporting a significant association between constipation and anxiety.

Anxiety and constipation likely form a bidirectional relationship with both “top-down” and “bottom-up” interactions. Anxiety can exacerbate constipation by altering autonomic nervous system function, impacting gut motility, and dysregulating the hypothalamic-pituitary-adrenal (HPA) axis (24, 25). It can also increase muscle tension in the pelvic floor, interfering with defecation (26). Additionally, anxiety-related coping mechanisms, such as hypervigilance, may worsen the perception of constipation (27). Conversely, chronic constipation, with symptoms like discomfort and bloating, can heighten stress and contribute to anxiety (28). Furthermore, changes in gut microbiota in constipation may influence neurotransmitter production, potentially exacerbating anxiety (29). This bidirectional relationship suggests that both conditions amplify each other’s severity.

Mental illnesses, particularly depression, are considered significant factors that increase the risk of anxiety (30). Studies have found a significant association between constipation and depression (31). However, the impact of depression as a key covariate on the strength of the association between constipation and anxiety remains unclear. In Model 3 of this study, after adjusting for depression, the association between constipation and anxiety remained statistically significant (OR: 1.33, 95% CI: 1.02-1.73, *P* = 0.038). Nonetheless, the strength of the association after adjusting for depression was significantly lower than that in the unadjusted model (OR: 2.01, 95% CI: 1.36-2.16, *P* < 0.001). These findings suggest that although depression may affect the strength of the association between constipation and anxiety, it does not negate the overall significance of this association.

The link between constipation and anxiety may involve the interaction between the central nervous system and the gastrointestinal tract. Key mechanisms include: 1. Brain-Gut Axis Dysfunction: Increased brain connectivity in the orbitofrontal cortex and thalamus in patients with functional constipation and anxiety/depression correlates with their symptoms (32). 2. Corticotropin-Releasing Factor (CRF): CRF affects bowel habits and gastric emptying via autonomic dysfunction. Anxiety disorders involve CRF pathway hyperactivity, making CRF receptors potential targets for treatment (33). 3. Gut Microbiota: Anxiety patients have altered gut microbiota, with reduced probiotics and increased pathogens, affecting gut hormone secretion and function (34). 4. Psychological Symptoms and Muscle Tension: Anxiety increases pelvic floor muscle tension, leading to dyssynergia. Constipation patients without physiological issues may have more psychological symptoms than those with colonic transit delay (35).

To address the potential causal relationship between constipation and anxiety, we conducted a MR analysis. Using genetic data from large-scale GWAS datasets, we found no significant causal association between constipation and anxiety. This suggests that while the observed association between constipation and anxiety in our cross-sectional analysis is robust, it may not reflect a causal relationship. These findings imply that genetic factors associated with constipation do not directly contribute to the development of anxiety. However, the absence of a causal link in MR does not rule out the possibility of a bidirectional relationship or a non-genetic mechanism influencing both conditions simultaneously. It is important to note that the NHANES study involved US participants, while the MR study primarily included Europeans. Population differences may contribute to result inconsistencies, as genetic, environmental, and cultural factors can affect the constipation-anxiety relationship. Previous studies have similarly identified population mismatches as a potential source of such inconsistencies (36, 37). This underscores the need for more diverse, population-specific studies to better understand the relationship between constipation and anxiety.

This study has several limitations. As a cross-sectional study, it cannot determine causality. Both constipation and anxiety were self-reported, potentially leading to reporting bias. Additionally, the definition of constipation was based on bowel movement frequency, which might not capture all aspects of the condition. MR analysis, while a useful tool for assessing causality, also has limitations. These include the reliance on genetic instruments that may not fully capture the complex biological pathways linking constipation and anxiety, as well as the potential for population differences that could affect the generalizability of the findings. Future research should explore the causal relationship and underlying mechanisms between constipation and anxiety to provide scientific evidence for clinical interventions and public health policies.

## 5 Conclusion

Using NHANES data, this study found a significant association between constipation and anxiety. This association remained significant even after adjusting for various covariates, suggesting an independent relationship. However, MR analysis did not identify a significant causal relationship between constipation and anxiety, indicating that while the association is strong, it may not be causal. Future research should further investigate the causal relationship and underlying mechanisms to provide scientific evidence for clinical interventions and public health policies.

## Data Availability

The datasets presented in this study can be found in online repositories. The names of the repository/repositories and accession number(s) can be found below: Publicly available datasets were analyzed in this study. All data entered into the analysis were from NHANES, which is publicly accessible to all.
