# Supplementary material for: The association between constipation and anxiety: a cross-sectional study and Mendelian randomization analysis
Source: Front Psychiatry. 2025 Mar 31;16:1543692. doi: 10.3389/fpsyt.2025.1543692 (PMC11995435; doi:10.3389/fpsyt.2025.1543692)
Supplement: Supplementary file 1 [file SupplementaryFile1.docx]

**Supplementary Materials Files**

To: **The Association Between Constipation and Anxiety: A Cross-Sectional Study and Mendelian Randomization Analysis**

by Yingxuan Huang, Yubin Wang, Boming Xu, Yilin Zeng, Peizhong Chen, Yisen Huang, Xiaoqiang Liu


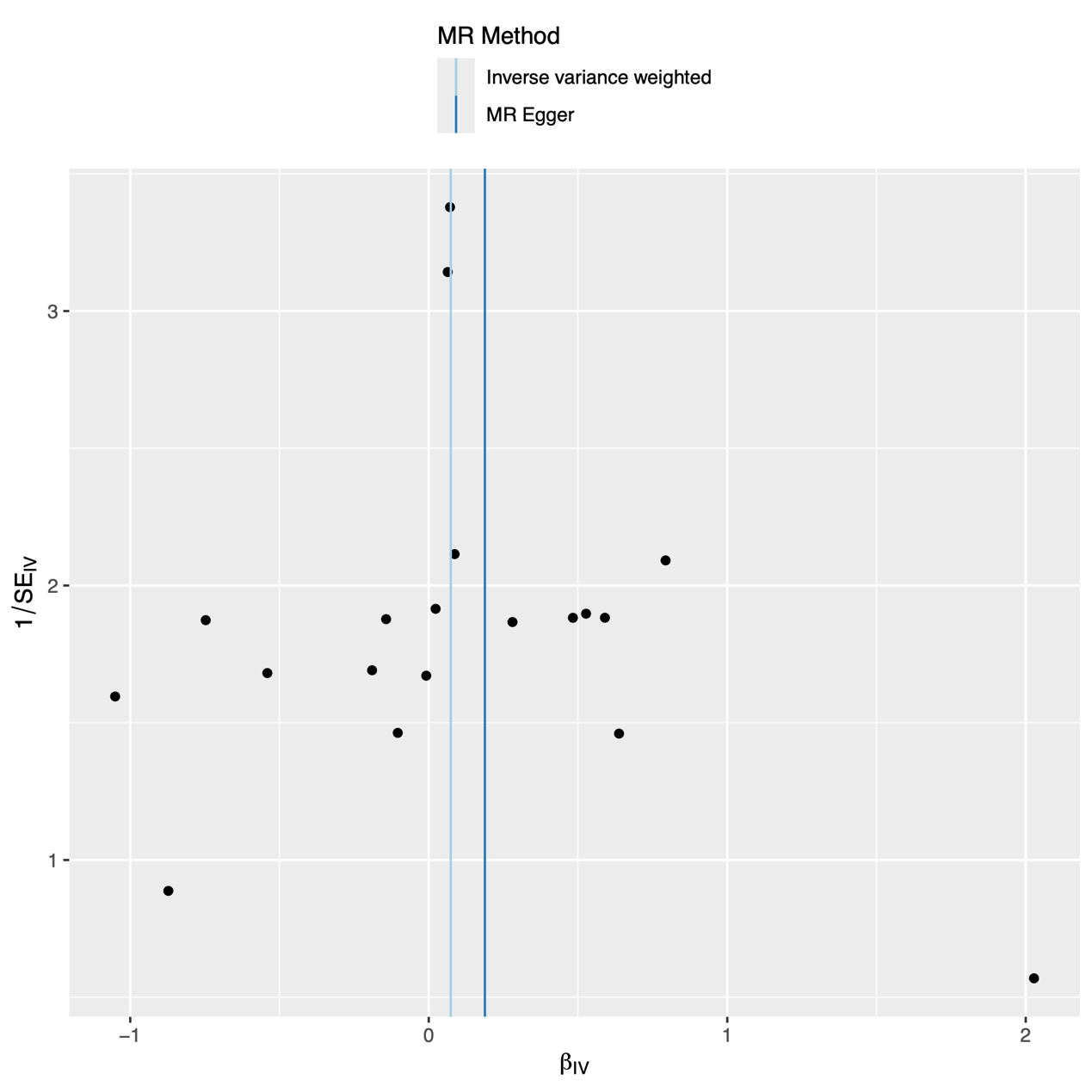


**Supplementary Figure 1 Funnel plot for assessing heterogeneity**


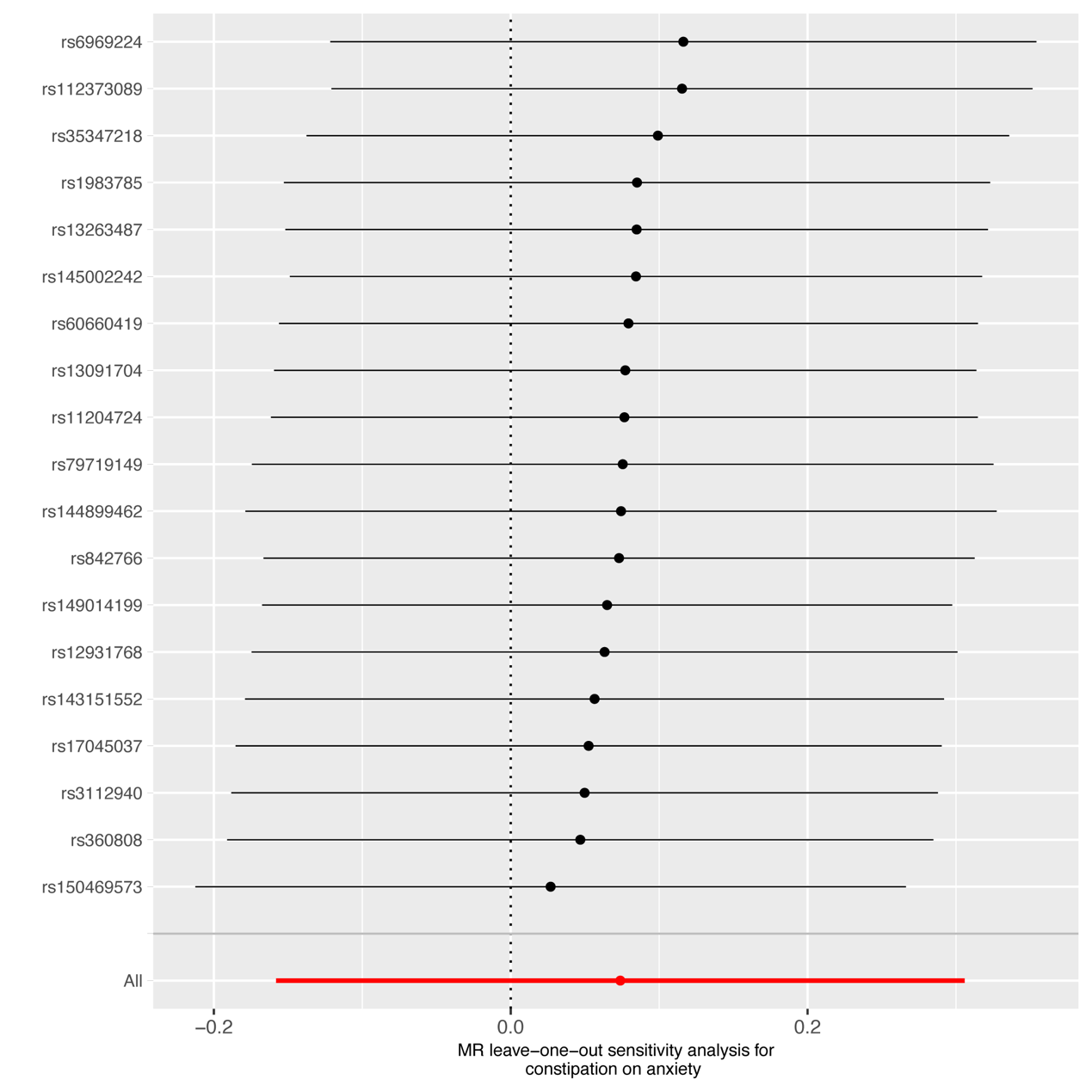


**Supplementary Figure 2 Leave-one-out plots for assessing the robustness**
